# Adolescent Catatonia Successfully Treated with Lorazepam and Aripiprazole

**DOI:** 10.1155/2014/309517

**Published:** 2014-08-12

**Authors:** Aaron J. Roberto, Subhash Pinnaka, Abhishek Mohan, Hiejin Yoon, Kyle A. B. Lapidus

**Affiliations:** ^1^Westchester Medical Center, Valhalla, NY 10595, USA; ^2^Old Dominion University, Norfolk, VA, USA; ^3^Icahn School of Medicine at Mount Sinai, New York, NY, USA

## Abstract

Catatonia is especially concerning in children and adolescents. It leads to significant impairment, including emotional distress, difficulty communicating, and other debilitating symptoms. In this case report, we discuss a patient with no previous history of neuroleptic medication or psychotic symptoms, presenting with first-episode catatonia in the presence of disorganized, psychotic thoughts. We then review the catatonia syndrome, citing examples in the literature supporting its underdiagnosis in children and adolescents, and discuss successful treatment modalities. It is important to diagnose and treat catatonia as efficiently as possible, to limit functional and emotional distress to the patient.

## 1. Introduction

Catatonia is a distinct syndrome of motor dysregulation, with symptoms including excessive and purposeless motor activity, alternating with immobility. It is a debilitating condition, leading to difficulty communicating, pronounced distress, and extreme discomfort. The condition is not uncommon among children, adolescents, and adults [1]. Catatonia has an approximate incidence of 0.6% for children and adolescents that are treated psychiatrically in an inpatient setting [2]. Unfortunately, catatonia is often inadequately recognized in children and adolescents since it is often overshadowed by medical, neurological, or developmental disorders [3, 4]. Careful consideration of the possibility of catatonic symptoms and targeted assessments when there is any reason for suspicion is critical to effectively diagnose and treat catatonia in the child and adolescent population. Benzodiazepines have been used to effectively treat catatonia, and these medications should be considered as a first-line treatment, especially given their limited adverse side effect profile [3]. In addition to benzodiazepines, ECT may be considered for those with catatonia, since it is also recognized as a preferred treatment [5].

## 2. Case Report

Herein we describe a 16-year-old neuroleptic naïve Caucasian male with past psychiatric history of ADHD and a family history including a mother with bipolar disorder, who presented to the pediatric ER with altered mental status. He had been assessed once in the pediatric ER, a year earlier, for alcohol intoxication but had no previously diagnosed medical conditions. Immediately before admission, he was lethargic and nonverbal, leading to transportation to a nearby ER, where he presented with macular rash over his cheeks and trunk, which resolved while he was waiting to be transferred to our facility.

Two days before admission, the patient reported to his mother that he was concerned about being attacked by his peers for vague and unclear reasons. In the following days, he gradually became alternatingly irritable, paranoid, easily agitated, disorganized, confused, and lethargic. He became increasingly stiff, with rigid posture and decreased oral intake of food and water. His mother was concerned that the patient's symptoms were caused by illicit substance ingestion, as the patient had stated that he tried phencyclidine (PCP) in the past. There were no reported periods of restlessness, pleasure seeking, goal-oriented behavior, grandiosity, delusional thoughts, or other symptoms suspicious for potential mania or psychotic disorder, prior to this episode. There were no previous or pending disciplinary school issues or legal actions. The patient had no known drug or environmental allergies. There was no history of catatonia, dystonia, stiffness, mutism, tic disorder, obsessive-compulsive disorder, or involuntary movements.

On presentation to our pediatric ER, the patient appeared suspicious, agitated, and confused. He was restless and not verbal nor otherwise communicative and was sitting up, hunched over with rigid, flexed, inwardly rotated extremities; he retained a stiff posture throughout the encounter. His eyes were at first closed although he was awake and responding to painful stimuli. He was initially confused, perplexed, and internally preoccupied. On physical examination, upper and lower extremities were hyperreflexic. Signs of cogwheeling or clasp-knife rigidity were not found on physical examination.

Blood alcohol and urine toxicology returned negative. The patient was then transferred to the pediatric medical inpatient unit. Computerized tomography, chest X-ray, and blood labs, including N-methyl-D-aspartate (NMDA) antibodies, all were within normal limits. Sedation was administered to manage agitation and lumbar puncture was performed; cerebrospinal fluid was sent for cell counts, cultures, and chemistries, which returned negative. Magnetic resonance imaging (MRI) was performed, under sedation due to agitation, on the second day of admission, and electroencephalogram was obtained, both of which were found to be normal.

On the second day of admission, lorazepam 1 mg intramuscularly (IM) was provided for continued agitation. After one dose, the patient became slightly more responsive to verbal commands, with a minimal decrease in rigidity for approximately 30 minutes, though he remained disoriented. Three hours later, after another dose of lorazepam 1 mg, rigidity decreased further and the patient became more lucid, able to move his head minimally in response to sounds and commands, though in a very slow and repetitive, stereotyped manner. His presentation and response to lorazepam, with negative workup, led to the diagnosis of catatonia by the psychosomatic medicine team consulting to the pediatric medical unit. On day 3, a standing dose of lorazepam, beginning with 0.5 mg daily, was initiated. He also received additional doses, as needed, for continued rigidity and stiffness. After each dose he became less rigid and his extremities were progressively less flexed, though he remained internally preoccupied and only able to respond minimally to commands. On day 5, the standing lorazepam dose was increased to 0.5 mg three times daily (TID), and the patient's mental status continued to improve; he became more lucid and better able to engage the interviewer. His fixed, flexed posture improved significantly, and he became able to weakly grasp the examiner's finger with his right hand (yet not with the left hand). He also was able to weakly and slowly move his toes bilaterally on command. He was hyperreflexic, with L3-L4 knee jerk and upper extremity reflexes rated 3+.

On day 6, the patient was minimally verbal and continued to have decreased oral intake. He had lost 13 pounds since admission, experienced enuresis in the morning, and had difficulty voiding while standing up. He remained paranoid and delusional, and autonomic changes became apparent with heart rate fluctuating between 48 and 106 beats per minute. At this time, lorazepam was increased to 1 mg TID. Over the next two days, he substantially improved, began eating meals, and became more verbally interactive and lucid. Rigidity and ambulation improved. Vital signs also stabilized though the patient remained guarded with suspicious affect and limited eye contact, shying away and pointing at the examiner. Aripiprazole was initiated at 2 mg once daily for treatment of ongoing disorganization, suspiciousness, and paranoia. Aripiprazole was chosen due to its known safety profile and clinical effectiveness in treating psychosis and catatonia, as evidenced by multiple case reports with effective results in the child and adolescent population [6–9]. In addition, aripiprazole treatment poses a lower risk for cardiac and metabolic effects, as well as hyperlipidemia, compared to other second generation neuroleptics [10], and has a long half-life allowing for once daily dosing. Lorazepam was continued at the current dose with ongoing improvement. Oral liquid intake improved and after 2 more days of treatment, the patient was less mute, less rigid, and less catatonic. His motor skills also improved dramatically. He was able to verbalize his thoughts effectively and no longer displayed restricted or slowed, stereotypical movements. As catatonic symptoms improved, he began to complain of generalized body ache, presumably due to muscular stiffness and rigidity, which was successfully treated with tylenol as needed. The patient continued to experience improvement of stiffness and rigidity, yet vague, paranoid thoughts and guardedness persisted. Abilify was titrated up to 10 mg for improved treatment of symptoms. On the tenth day of hospitalization, he was discharged home on aripiprazole 10 mg daily and small dose of lorazepam with taper instructions and with a scheduled follow up psychiatry appointment.

Lorazepam was slowly tapered over the course of four weeks after discharge in order to continue to target lingering stiffness and rigidity, which completely resolved with treatment. As of 2 months after discharge from the pediatrics unit, he returned as outpatient and positive psychotic symptoms, rigidity, and stiffness had completely resolved. There was a mild degree of negative symptoms present, including slow processing speed, constricted affect, fatigue, and decreased concentration, yet the catatonia had completely resolved.

## 3. Discussion

Catatonia is a syndrome of motor dysregulation that includes excessive motoric activity, stereotypical movements, extreme negativism or mutism, echolalia or echopraxia, and other involuntary movements [1, 6, 11, 12]. Catatonia has been associated with a wide variety of psychiatric, medical, neurological, substance-related, endocrine, infectious, and metabolic conditions [13]. In psychiatry, it is traditionally linked to schizophrenia, though catatonia is not uncommon in mood disorders [14–16]. In a significant minority, no cause is identified [15].

Catatonia has been previously reported to be rare in child and adolescent patients [2]. However, recent evidence suggests that the prevalence in this population is similar to the adult population [4].

Benzodiazepines and electroconvulsive therapy (ECT) are considered first-line treatments, and most patients respond well when adequately treated [5]. Complete remission of catatonia has been reported in 70–80% of cases following administration of a benzodiazepine alone [17–20]. ECT is recommended when there is insufficient improvement with benzodiazepines [17, 21].

Antipsychotics are generally not recommended during acute catatonia, as they can increase the risk of precipitating symptoms including neuroleptic malignant syndrome (NMS) or akinesia [22]. Dysregulation between motor/premotor cortex and the basal ganglia may contribute to this risk; in NMS striatal dopamine D2 receptor blockade may impact this motor feedback pathway, and in catatonia, GABAergic dysfunction may play a role [22, 23]. It has been postulated that dopamine D2 hypoactivity, glutamate NMDA hyperactivity, and GABAa hypoactivity contribute to the development of catatonia [23] and that lorazepam and other drugs that affect GABAa activity (e.g., anticonvulsants) exert their beneficial effect and resolution of catatonia through GABA modulation [19].

Despite the risk of akinesia, antipsychotics may be effective in treatment-resistant catatonia and catatonia with psychosis [7, 24–26]. For treatment of residual psychotic symptoms, second generation antipsychotics with low D2 blockade (quetiapine, olanzapine) or with D2 partial agonism (aripiprazole) are preferred [8]. These drugs may also treat the underlying psychopathology, such as a manic or psychotic episode [27]. Successful treatment of catatonia with aripiprazole in an adolescent with psychosis has been reported [6, 10]. Olanzapine has also shown efficacy in the treatment of catatonic schizophrenia, both in children [28], and adolescents [29, 30]. Treatment of catatonia in adolescents with amantadine, a weak NMDA antagonist with dopaminergic, cholinergic, and serotonergic activity, has also demonstrated efficacy [22, 31]. In one study, 7 patients were treated with clozapine after unsuccessful trials of lorazepam and other atypical antipsychotics, and 6/7 (86%) experienced a beneficial effect following slow titration, a good overall response for treatment of catatonia [25]. In a large case series including 39 patients with catatonia, 61.5% of patients were on quietapine at the time of catatonia recovery and 51.3% of patients were on quietapine when discharged versus only 17.9% of patients on quietapine on admission [26].

Our patient experienced a first-episode of catatonia and psychosis, with disorganized and paranoid thought process. Consistent with previous reports, lorazepam rapidly relieved our patient's catatonic symptoms. Lorazepam challenge has been found to be effective in diagnosing and treating catatonia; transient improvement of catatonia following lorazepam administration is suggestive of eventual benefit after completing the treatment course [7]. Aripiprazole has shown efficacy in treating both psychosis and refractory catatonia in the long term [6–9], and as mentioned previously, has a lower risk of metabolic and cardiac effects in the child and adolescent population [10]. In this case, the administration of lorazapam was effective in treating our patient's catatonia, while delusional and paranoid thoughts were relieved by concomitant treatment with aripiprazole, which has also been effective in treating catatonia based on cases in the literature. Here, catatonia was diagnosed efficiently, which facilitated successful treatment, beginning with lorazepam administration. Clinicians should maintain a low threshold for initiating benzodiazepine treatment for children and adolescents experiencing first episode catatonic symptoms.
